# Diet-Induced Obesity and Ghrelin Effects on Pituitary Gonadotrophs: Immunohistomorphometric Study in Male Rats

**DOI:** 10.22074/cellj.2016.3843

**Published:** 2016-01-17

**Authors:** Natasa Ristic, Darko Stevanovic, Dejan Nesic, Vladimir Ajdzanovic, Rastko Rakocevic, Ivana Jaric, Verica Milosevic

**Affiliations:** 1 Institute for Biological Research Sinisa Stankovic, University of Belgrade, Belgrade, Serbia; 2 Institute of Medical Physiology, School of Medicine, University of Belgrade, Belgrade, Serbia

**Keywords:** Obesity, Ghrelin, Gonadotrophs, Male, Rats

## Abstract

**Objective:**

The close relationship between energy metabolism, nutritional state, and reproductive physiology suggests that nutritional and metabolic disorders can disrupt normal reproductive function and fertility. Considering the importance of leptin and ghrelin effects in regulation of the hypothalamic-pituitary-gonadal axis, the objective of this study was to investigate the influence of obesity and centrally applied ghrelin on immunohistochemical appearance and quantitative morphology of the pituitary follicle-stimulating hormone (FSH) and luteinizing hormone (LH) producing cells in adult male rats.

**Materials and Methods:**

In this experimental study, animals were given two differ- ent diets: normal-fat (NF) and high-fat (HF), for 4 weeks, corresponding to normal and positive energy balance (n=2×14), respectively. Each group was subsequently divided into two subgroups (n=7) receiving intracerebroventricular (ICV) injections of either ghrelin [G, 1 µg/5 µL phosphate buffered saline (PBS)] or vehicle (5 µL PBS, control group) every 24 hours for five consecutive days.

**Results:**

Morphometric analyses showed that in HF control group, the percentage of FSH cells per unit volume of total pituitary gland tissue (in μm^3^), i.e. volume density (Vvc), was increased (P<0.05) by 9.1% in comparison with the NF controls. After ICV treatment with ghrelin, volume (Vc) and volume density (Vvc) of FSH cells in ghrelin+NF (GNF) and ghrelin+HF (GHF) groups remained unchanged in comparison with NF and HF controls. Volume of LH cells in HF control group was increased by 17% (P<0.05), but their Vvc was decreased by 8.3% (P<0.05) in comparison with NF controls. In GNF group, the volume of LH cells increased by 7% (P<0.05), in comparison with the NF controls, but in GHF group, the same parameter remained unchanged when compared with HF controls. The central application of ghrelin de- creased the Vvc of LH cells only in GNF group by 38.9% (P<0.05) in comparison with the NF control animals.

**Conclusion:**

The present study has shown that obesity and repetitive ICV administra- tion of low doses of ghrelin, in NF and HF rats, modulated the immunohistomorphometric features of gonadotrophs, indicating the importance of obesity and ghrelin in regulation of the reproductive function.

## Introduction

The global increase in the prevalence of energy metabolism disorders and diseases that arise from them indicated the importance of understanding the energy balance, which is defined as the perfect equilibrium between energetic intake and expenditure. It is well known that the energy homeostasis is important in controlling the synthesis and secretion of a number of hormones and any disturbance in this respect is reflected in a change in the activity of numerous organ systems and endocrine axes. The ability to maintain equilibrium of the energy balance and the ability to reproduce are essential for survival and maintenance of the species. The relationship between energy metabolism, nutritional state, and reproductive physiology (1,2) suggests that nutritional disorders (obesity, malnutrition and anorexia nervosa) and metabolic disturbances can disrupt normal reproductive function and fertility. Ghrelin and leptin, primary signals in the regulation of food intake (3), operate as endocrine–paracrine mediators linking energy homeostasis and reproduction (1). The central nervous system, especially the hypothalamus and pituitary, are the primary sites of action of these mediators of appetite, whereby they influence gonadotropinreleasing hormone (GnRH) pulsatility as well as follicle-stimulating hormone (FSH) and luteinizing hormone (LH) production and secretion. 

Ghrelin is a growth-hormone (GH)-releasing peptide, which is predominantly synthesized by the gut and have wide array of biological functions, including the signaling of energy insufficiency and energy homeostasis (3,5). Some studies also suggest that ghrelin may contribute in the control of key aspects of reproduction (6,7), by modulation of the hypothalamic-pituitary-gonadal function. Ghrelin seems to operate as an inhibitory signal for the gonadotropic system in different species, acting via hypothalamic and pituitary receptors (8,9). As a both central and peripheral signal of reduced energy reserves, ghrelin affects on the complex interplay of gonadotropins and gonadal hormones, which are essential for fertility (10,11). 

Considering the importance of the central ghrelin effects in regulation of the hypothalamic-pituitary-gonadal axis, the objective of this study was to investigate the influence of obesity and centrally applied ghrelin on immunohistochemical appearance and quantitative morphology of the pituitary FSH and LH cells in adult male rats. 

## Materials and Methods

### Experimental design

In this experimental study, animals were given two different diets: normal-fat (NF) and high-fat (HF), for 4 weeks, corresponding to normal and positive energy balance (n=2×14), respectively. Each group was subsequently divided into two subgroups (n=7) receiving intracerebroventricular (ICV) injections of either ghrelin [G, 1 µg/5 µL phosphate buffered saline (PBS)] or vehicle (5 µL PBS, control group) for five consecutive days. This schedule was chosen based on earlier results that showed that such a regime was sufficient to induce significant hormonal, metabolic and behavioral changes (12,13). Two experiments were performed, a preliminary one with three rats in each of four groups and a larger confirmatory study with seven rats per group. 

## Animals and food regimens

This original research involved male Wistar rats bred at the "Galenika" Institute of Biomedical Research in Belgrade, Serbia. They were kept in individual metabolic cages under a 12:12 hour light– dark cycle, at 22 ± 2˚C, and were accustomed to daily handling. At 4 weeks of age, the rats were randomly divided into two groups, each subjected to a different dietary regime for the next 4 weeks. Namely, NF animals received the standard balanced diet for laboratory rats ad libitum (D.D. Veterinarski Zavod Subotica, Subotica, Serbia). The HF group received ad libitum the standard diet enriched with 30% lard (14). Water was freely available to all rats. 

## Animal preparation and treatment

After 4 weeks on dietary regime, the animals were anesthetized with intramuscular ketamine (50 mg/ kg, Pfizer, USA), and xylazine (80 mg/kg, Bayer AG, Germany). Each was equipped with a headset for ICV injection, consisting of a silastic-sealed 20-gauge cannula positioned in the right lateral cerebral ventricle (1 mm posterior and 1.5 mm lateral to the bregma, and 3 mm below the cortical surface) (15). A small stainless steel anchor screw was placed at a remote site on the skull. The cannula and screw were cemented to the skull with standard dental acrylic. After surgery, the animals received a single dose of 0.28 mg/kg buprenorphin (Buprenex, Reckitt Benckiser Healthcare, Germany) subcutaneously (SC) followed by a recovery period of 1 week. Each group was divided into two subgroups, one treated ICV with 1 µg of ghrelin (Global Peptide Services, LLC, USA) in 5 µl PBS every 24 hours for 5 consecutive days, while the other was a control group receiving 5 µl of PBS. The ICV treatments were administered between 10:00 and 11:00 am, and all animals were killed by decapitation under deep isoflurane anesthesia, 2 hours after the last ICV injection. After excision, the pituitaries were weighed, fixed in Bouin’s solution for 48 hours and embedded in Paraplast wax. The ratio of the measured pituitary weight and the body weight for each animal provided the basis for the relative pituitary weight calculation. All animal procedures were in compliance with the Environment European Commission (EEC) Directive (86/609/EEC) on the protection of animals used for experimental and other scientific purposes, and were approved by the Ethical Committee for the Use of Laboratory Animals of the Institute for Biological Research "Sinisa Stankovic", University of Belgrade, Serbia. 

## Immunohistochemical studies

Gonadotrophs (FSH and LH cells) were localized immunohistochemically using the peroxidase-antiperoxidase method. Polyclonal rabbit anti-rat beta-FSH (βFSH, 1:300 v/v) and polyclonal rabbit anti-rat beta-LH (βLH, 1:500 v/v) served as primary antibodies. Antisera to rat βFSH and βLH were obtained from Dr. A. F. Parlow, National Hormone Peptide Program (NHPP), Harbor-UCLA Medical Centre, USA. The antisera specificities were assessed by the National Institute of Diabetes and Digestive and Kidney Diseases (NIDDK). Sections were incubated in primary antibodies for 45 minutes at room temperature. After washing in PBS (pH=7.4), sections were incubated for 1 hour with polyclonal swine anti-rabbit immunoglobulins conjugated with horseradish peroxidase (IgG/HRP, Dako A/S, Denmark). The antigen– antibody complex was visualized by incubating the sections with a chromogen substrate, 0.05% 3,3-diaminobenzidine (DAB, Dako A/S, Denmark) and 0.03% H_2_O_2_. The incubated sections were counterstained with hematoxylin. Control sections were incubated with PBS without primary antisera.

## Morphometry

Morphometrical measurements were performed as described previously (16-18). Namely, two pituitary sections (5 μm thick) from the superior, three from the middle and two from the inferior part (seven horizontal sections, 20 μm apart in total) of the rat pituitary glands were analysed. The point counting method was used at an overall magnification of ×1000 (19). The M42 multipurpose test grid, inserted into the ocular of a Zeiss light microscope (Jena, Germany), was randomly placed on the pituitary section at the beginning of counting. Counting was carried out on the following 50 test fields per section. Average values were calculated per pituitary i.e. per animal (7 sections, 350 test fields) and five pituitaries were analyzed per group. Cell volume (Vc, μm^3^), volume of the nuclei (Vn, μm^3^) and volume density (Vvc, percentage of immunoreactive cells in μm^3^, %) were determined for FSHor LH-immunoreactive cells.

The following parameters were counted: Pn; number of points hitting on nuclei of immuno-histochemically labeled cells inside the test field, Ptc; number of points hitting on cytoplasm of immunohistochemically labeled cells inside the test field, Nn; number of immuno-histochemically labeled cell nuclei inside the test field.

The formula for calculating the nuclei volume was: Vn=VVnNV that for the cell volume calculation was: Vc=1NV , and where V_V_n__; the volume density of FSH or LH cell nuclei and N_v_; the numerical density of FSH or LH cells. V_V_n__ provides information about nuclei attendance in the estimated cells and is calculated as follows: VVn=ΣPnΣPtc.

Since rat FSH and LH cells are mononuclear, N_V_ corresponds to the number of cells per cubic millimetre, according to the formula:

NV=(kβ)(NA32VVn12)

On the basis of earlier karyometric studies (20), the shape coefficient β for pituitary cells was estimated to be 1.32, where k is a factor related to cell distribution according to their size (in the case of FSH and LH cells, its value is 1) and N_A_ is the number of cells in the plane of the pituitary tissue section. N_A_ is calculated as follows: NA=ΣNnΣPtc a, where a represents the rhombic area belonging to every point of the test system and is calculated using the formula: a=d23122 , where d is the test line length in the test system employed.

Vvc is calculated as the ratio of the sum of Pn and Ptc (Pn+Ptc) and the total number of points in the test system. Since the test system with 42 points was used and parameters were calculated using 50 test fields, the definite formula was: Vvc=(Pn+Ptc)50*42.

Digital images were made using a DM RB Photomicroscope (Leica, Germany) with a JVC TK 1280E Video Camera (Leica, Germany). The Qwin program (Leica, Germany) was used for image acquisition.

## Statistical analyses

Statistica^®^ version 5.0 (StatSoft, Inc., Tulsa, OK, USA) was used for all statistical analyses. Morphometric data obtained for the experimental groups were subjected to one-way ANOVA. Duncan’s multiple range test (Pharmacological Calculation System, 1986) was used for post hoc comparisons between groups. The confidence level of P<0.05 was considered statistically significant. The data are presented as means ± SD.

## Results

### Body and pituitary weights

Data for body weights and absolute and relative pituitary weights are summarized in table 1. Body weight in the HF control group was significantly increased by 47.4% (P<0.05) compared to the NF controls. In both groups receiving ICV injection of ghrelin (GNF and GHF), body weights were significantly increased (P<0.05) by 8.4 and 5.7%, respectively, in comparison with NF and HF controls. Absolute pituitary weight in HF control rats was significantly increased (P<0.05) by 35.9% in comparison with NF controls, but not significantly changed in ghrelin treated animals (GNF and GHF) compared to the NF and HF controls. Relative pituitary weight was not significantly changed in any of the examined group (Table 1).

### Immunohistochemical findings

**Fig.1:**
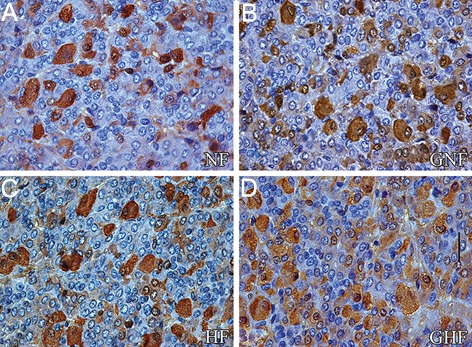
A, B. Immunoreactive FSH cells in the pars distalis of the pituitary gland from NF and HF control, C and D. From ghrelin (GNF and GHF) treated rats (scale bar=16 μm). FSH; Follicle-stimulating hormone, NF; Normal-fat, HF; High-fat, GNF; Ghrelin+NF and GHF; Ghrelin+HF.

The FSH and LH cells of adult control males were predominatly oval in shape with prominent, often eccentrically located nuclei. They were strongly immunohistochemically stained and positioned throughout the pituitary pars distalis, alone or in groups, often in close contact with blood capillaries (Fig .1A).

Histological analysis of FSH cells showed that obesity and ICV treatment with ghrelin did not affect the histological features of FSH cells in the examined groups (Fig .1B-D).

**Fig.2:**
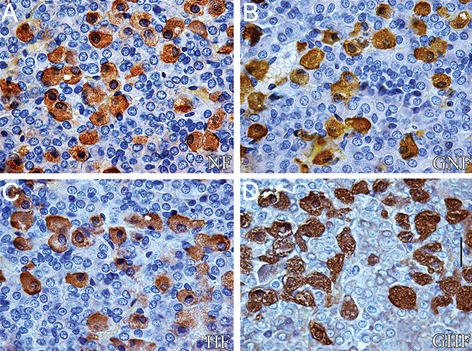
A, B. Immunoreactive LH cells in the pars distalis of the pituitary gland from control NF and HF rats, C and D. From ghrelin (GNF and GHF) treated rats (scale bar=16 μm). LH; Luteinizing hormone, NF; Normal-fat, HF; High-fat, GNF; Ghrelin+ NF and GHF; Ghrelin+HF.

LH cells in the HF control group were larger and less numerous than in NF control group (Fig. 2A, B). After ICV treatment with ghrelin, the same type of change in GNF group was noticed (Fig .2C). In the GHF group, ghrelin did not affect the histological features of LH cells (Fig .2D) when compared to the HF controls (Fig .2B).

**Table 1:** Body weights and absolute and relative pituitary weights after 5 daily intracerebroventricular (ICV) ghrelin injections to male rats on different diets


Groups	Body weight (g)	Absolute pituitary weight (mg)	Relative pituitary weight (mg/%)

Control NF	215.1 ± 11.4	7.8 ± 0.7	3.5 ± 0.5
Ghrelin NF	233.1 ± 15.1^b^ (+8.4%)	7.7 ± 0.7 (-2.0%)	3.4 ± 0.4 (-2.8%)
Control HF	317.0 ± 19.3^a^ +47.4%)	10.6 ± 1.0^a^ (+35.9%)	3.3 ± 0.5
Ghrelin HF	335.0 ± 12.5^c^ (+5.7%)	9.7 ± 1.1 (-8.5%)	2.9 ± 0.6 (-12.1%)


NF; Normal fat, HF; High fat, GNF; Ghrelin NF, GHF; Ghrelin HF, ^a^; P<0.05 HF vs. NF control rats, ^b^; P<0.05 GNF vs. NF and ^c^; P<0.05 GHF vs. HF. Data presented as mean ± SD (n=7).

### Morphometric findings

**Fig.3:**
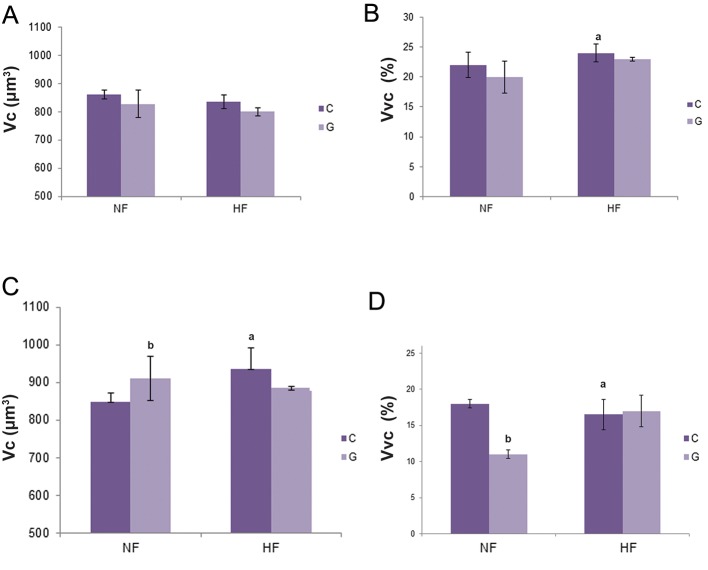
A, C. Cellular volume (Vc, μm^3^) of FSH and LH cells, B and D. Relative volume density (Vvc, %) of FSH and LH cells expressed as a percentage of total gland tissue (in μm^3^). All values are means ± SD, n=7, P<0.05. C; Control animals, G; Ghrelin treated animals, FSH; Folliclestimulating hormone, LH; Luteinizing hormone, NF; Normalfat, HF; High-fat, GNF; Ghrelin+NF, GHF; Ghrelin+HF, a; NF controls vs. HF controls and b; GNF vs. NF controls,

Morphometric analyses showed that in HF control group, the Vc of FSH was not changed, but the percentage of FSH cells per unit volume of total pituitary gland tissue (in μm^3^) i.e. Vvc was increased (P<0.05) by 9.1% in comparison with the NF controls. After ICV treatment with ghrelin, Vc and Vvc of FSH cells in GNF and GHF groups remained unchanged (Fig .3A, B).

Volume of LH cells in HF control group was increased by 17% (P<0.05), but their Vvc was decreased by 8.3% (P<0.05) in comparison with NF controls. In GNF group, the volume of LH cells increased by 7% (P<0.05), in comparison with the NF controls, but in GHF group, the same parameter remained unchanged when compared with HF controls. The central application of ghrelin decreased the Vvc of LH cells only in GNF group by 38.9% (P<0.05) in comparison with the NF control animals (Fig .3C, D).

## Discussion

Using the immunohistomorphometric approach, this study showed that obesity and ghrelin cause some moderate inhibiting effects on gonadal axis at the pituitary level, after 5 consecutive daily central injections. 

Diet-induced positive energy balance led to the expected increase in body weight in HF when compared to the NF control animals, followed by increased absolute pituitary weight. Considering that the relative weight of pituitary gland was not changed, we can indicate that increased absolute pituitary weight reflects the body weight change. However, it is well known that obesity disrupts reproductive function both in experimental animals (21) and in humans (22). Thus, in obesity, plasma levels of leptin, anorexigenic adipose tissue-derived hormone, are elevated (14). As leptin plays the major role in interactions between nutritional status of the body and the function of hypothalamic-pituitary-gonadal axis (3,23), it may stimulate the production and release of FSH and LH. Conversely, it may also have inhibitory effects at high concentrations originating from obesity (24). 

Under our experimental conditions morphometric analysis of immunopositive gonadotrophs revealed increased Vvc of FSH cells, as well as increased Vc and reduced Vvc of LH cells in HF rats, when compared to NF control animals. The increase in LH cell volume (Vc) may be a consequence of reduced release of LH, which has already been described in obese males (25). In our experimental conditions, obesity also changed the dynamics of pituitary gonadotrope cell populations. Namely, Vvc of FSH cells increased, while Vvc of LH cells decreased in HF rats in comparison with NF control animals. This result could be explained by transdifferentiation within the pituitary cell populations (26). To be precise, Childs (26) observed cells that contain adrenocorticotropic hormone (ACTH) and LH, FSH, thyrotropin (TSH) or prolactin (PRL) hormonal content, which indicated that they were multipotential and had the capacity to augment or reduce the output of some kind of pituitary cells, to maintain total homeostasis regardless of the changes in energy status. Literature data indicate reduced FSH and LH serum concentrations determined in HF male rats (27), which is considerably compatible with our immunohistomorphometric findings in the milieu of obesity, as well as with the subsequent leptin resistance related hypogonadotropic hypogonadism found in overweight and obese males (28). 

Central ghrelin treatment significantly increased body weight in both examined experimental groups when compared with saline treated controls, which is in accordance with earlier findings from our and other laboratories (13,29), and confirms the wellknown orexigenic effect of this (acylated) form of ghrelin. However, central ghrelin treatment did not significantly affect either absolute or relative pituitary weight in any group, in comparison with the corresponding controls. 

It has been shown that suppression of circulating gonadotropin levels after acute and chronic elevation of ghrelin concentrations delays onset of puberty (30,31). Also, ghrelin exerts strong inhibitory effect on embryo implantation and development (6). In adulthood, systematic or intracerebral administration of ghrelin can induce significant inhibitory responses in LH, and to a much less extent in FSH secretion in rodents, sheep, non-human primates and humans (31). 

In our study, immunohistomorphometric parameters of FSH cells in GNF and GHF groups after central ghrelin treatment remained unchanged, in comparison with the corresponding controls. Our results are consistent with previous study Fernández-Fernandez et al. (32), which showed that FSH secretion *in vivo* was apparently independent from ghrelin action. Also, in humans, ghrelin is unable to control FSH secretion (33). Herein, central ghrelin treatment increased volume of LH cells (Vc) and decreased their volume density (Vvc) in GNF group in comparison with the corresponding controls. These results may indicate reduced LH secretion with the potential decrease in LH serum concentrations. Furuta et al. (34) suggest that ghrelin exerts a profound suppressive influence on pulsatile LH secretion. The inhibitory effect of ghrelin on LH secretion observed *in vivo* can be explained by the decrease of LH response to LH-releasing hormone (LHRH) detected *in vitro*. Namely, the suppressive effect of ghrelin is more potent after gonadectomy, when LHRH release is increased. 

In contrast to its effects *in vivo*, ghrelin stimulates FSH and LH secretion *in vitro*, but the mechanism involved in this effect remains unknown (32). 

The lack of effect of ghrelin on LH cells in the HF group of animals could be explained by relatively low doses of centrally administrated ghrelin and/or by pattern of delivery (injections vs. infusions), as numerous studies have shown that the dosage regimen and experimental approach change the degree of inhibitory influence of ghrelin on LH cells (32,34,35). 

## Conclusion

The present study has shown that repetitive ICV administration of low doses of ghrelin, in normally and HF rats, modulated the immunohistomorphometric features of gonadotrophs cells, indicating the importance of ghrelin in regulation of the reproductive function. Ghrelin and leptin can be considered as the hormonal signals with opposite effects on reproductive axis linking energy balance and reproductive function, two the most important factors for the survival and evolutionary advancement of mammals. 
