# Diet-Induced Obesity and Ghrelin Effects on Pituitary Gonadotrophs: Immunohistomorphometric Study in Male Rats

**DOI:** 10.22074/cellj.2016.4326

**Published:** 2016-05-30

**Authors:** Natasa Ristic, Darko Stevanovic, Dejan Nesic, Vladimir Ajdzanovic, Rastko Rakocevic, Ivana Jaric, Verica Milosevic

**Affiliations:** 1 Institute for Biological Research Sinisa Stankovic, University of Belgrade, Belgrade, Serbia; 2 Institute of Medical Physiology, School of Medicine, University of Belgrade, Belgrade, Serbia

In this article which was published in Cell J, Vol 17, No 4, Jan-Mar (Winter) 2016, on pages 711-719, the name of the all authors were published incorrectly as: "Ristic Na- tasa, Stevanovic Darko, Nesic Dejan, Ajdzanovic Vladimir, Rakocevic Rastko, Jaric Ivana, Milosevic Verica". The correct one is "Natasa Ristic, Darko Stevanovic, Dejan Nesic, Vladimir Ajdzanovic, Rastko Rakocevic, Ivana Jaric, Verica Milosevic".

This correction was requested by the corresponding author.

